# Specific Spoilage Bacterium of Chilled Sturgeon Fillets and the Bacterial Reduction Effect of ClO_2_

**DOI:** 10.1002/fsn3.71051

**Published:** 2025-10-26

**Authors:** Shirui Yu, Qin Cen, Shan Yu, Wenkang Hu, Xuechun Yin, Min Shan, Xuefeng Zeng, Xiaohua Chen

**Affiliations:** ^1^ Moutai Institute Renhuai China; ^2^ College of Life Science Guizhou Univerity Guiyang China; ^3^ College of Life Sciences Hengyang Normal University Hengyang China

**Keywords:** chlorine dioxide, specific spoilage bacteria, sturgeon fillet

## Abstract

Chilled sturgeon fillets are highly perishable, posing a significant challenge to their long‐distance transportation. This study aimed to identify the dominant spoilage bacteria in refrigerated sturgeon fillets and evaluate the effectiveness of chlorine dioxide (ClO_2_) treatment in inhibiting bacterial growth and preserving product quality. The results indicated that spoilage microorganisms proliferated rapidly during storage, with sensory, physicochemical, and microbiological parameters reaching critical thresholds by the 10th day. High‐throughput 16S rDNA sequencing revealed seven dominant spoilage bacteria: *Rahnella aquatilis* , *Pseudomonas fluorescens* , *Pseudomonas jessenii* , *Citrobacter freundii* , *Chryseobacterium indologenes*, *Hafnia alvei* , and *Serratia fonticola* . Among them, *Pseudomonas jessenii* was identified as the specific spoilage bacterium with significant spoilage activity. Using response surface methodology, the optimal ClO_2_ treatment parameters for inhibiting *Pseudomonas jessenii* were determined to be a ClO_2_ concentration of 36 μg/mL, a fillet thickness of 6 mm, and a soaking time of 17 min. The application of ClO_2_ effectively reduced spoilage bacterial loads, offering a promising strategy for extending the shelf life of chilled sturgeon fillets.

## Graphical abstract

This study aimed to identify the dominant spoilage bacteria in refrigerated sturgeon fillets and evaluate the effectiveness of chlorine dioxide (ClO₂) treatment in inhibiting bacterial growth and maintaining product quality. The 16S rDNA sequencing revealed seven dominant spoilage bacteria: *Rahnella aquatilis* , *Pseudomonas fluorescens* , *Pseudomonas jessenii* , *Citrobacter freundii* , *Chryseobacteriumin dologenes*, *Hafnia alvei* and *Serratia fonticola* . Using response surface methodology, the optimal ClO_2_ treatment parameters for inhibiting *Pseudomonas jessenii* were determined to be a ClO_2_ concentration of 36 μg/mL, a fillet thickness of 6 mm, and a soaking time of 17 min.

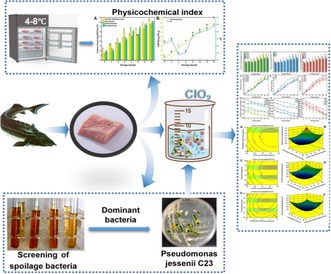

## 1 Introduction

Sturgeon is an ancient fish species whose products are characterized by a high‐quality protein profile, with an essential‐to‐nonessential amino acid ratio exceeding 60%. In addition, sturgeon is rich in trace elements essential to human health and contains various bioactive compounds, including glycosaminoglycans, chondroitin sulfate, and fatty acids. Its deep‐processed products are highly valued in the market (Chen et al. 2022). However, due to its abundant nutrients and high moisture content, sturgeon fillets are highly susceptible to microbial spoilage during transportation and storage. Spoilage bacteria can rapidly proliferate by utilizing the available nutrients, leading to the production of harmful metabolites (Tan et al. 2022). Even under ice‐temperature storage, problems such as freshness loss, discoloration, and texture deterioration persist, leading to food safety concerns and a significant reduction in commercial value. Therefore, it is crucial to identify and control the specific spoilage microorganisms responsible for the deterioration of sturgeon fillets during chilled storage.

During storage, specific spoilage bacteria can adapt to the environmental conditions and become dominant, producing various spoilage metabolites. Currently, the main spoilage microorganisms of low‐temperature frozen fish include *Photobacterium phosphoreum* , *Shewanella putrefaciens* , *Brochothrix thermosphacta* , *Pseudomonas* spp., *Aeromonas* spp., and *Lactic acid bacteria* (Jia et al. 2019; Li et al. 2020). The specific spoilage bacteria present in fish products vary depending on several factors, such as fish species, habitat water quality, fishing method, harvest season, storage conditions, and sampling site. Therefore, identifying the specific spoilage bacterium of sturgeon fillets under ice‐temperature and implementing targeted bacterial control strategies can effectively reduce spoilage and extend the shelf life of the product (Tan et al. 2022).

Chlorine dioxide (ClO_2_) is an effective, safe, and broad‐spectrum disinfectant that has been widely applied in the preservation of various foods, including aquatic products (Singh et al. 2020). Previous studies have demonstrated that ClO_2_ treatment, when combined with vacuum packaging, significantly suppresses protein and lipid oxidation, inhibits the growth of spoilage bacteria, and preserves the volatile flavor profile of pike samples during refrigerated storage (Du et al. 2022). ClO_2_ combined with slightly acidic electrolytic water treatment of large yellow croaker can inhibit microbial growth, lower pH value, malondialdehyde content, and total volatile basic nitrogen content compared to the control group, and increase water holding capacity. Moreover, this treatment delayed the degradation of umami amino acids and the accumulation of bitter amino acids in the fish samples (Lan et al. 2024).

In this study, the changes in physicochemical properties, sensory attributes, flavor, and the species and abundance of spoilage microorganisms in cultured sturgeon fillets during chilled storage were systematically analyzed. Dominant spoilage bacteria were isolated and identified. Subsequently, these dominant bacteria were re‐inoculated into sterile fish samples, and the specific spoilage organism was determined based on changes in TVB‐N levels and bacterial colony counts. The effects of ClO_2_ concentration, impregnation time, and fillet thickness on the spoilage bacteria and residual chlorine content were investigated. A response surface model was established to optimize the bacteriostatic treatment parameters targeting the specific spoilage bacterium in chilled cultured sturgeon fillets. The findings of this study provide a theoretical foundation for enhancing quality control throughout the sturgeon industry chain, reducing economic losses and food safety risks, and are expected to significantly improve both the economic value and social impact of the sturgeon industry.

## 2 Materials and Methods

### 2.1 Experimental Materials and Reagents

Sturgeon was supplied by Guizhou Jiaqi Aquaculture Foods Co. Ltd. (Guiyang, Guizhou, China). Broth medium, hydrogen peroxide, glucose, lactose, glucose peptone water medium, methyl red indicator, and hydrogen peroxide (Beijing Solarbio Science & Technology Co. Ltd., Beijing, China). Sodium hypochlorite and bromocresol purple (Tianjin Zhiyuan Chemical Reagents Co. Ltd., Tianjin, China). Bacterial genomic DNA extraction kit (Tiangen Biotech Co. Ltd., Beijing, China). All other chemicals used were of analytical grade unless otherwise specified.

### 2.2 Quality Changes in Farmed Sturgeon Fillets During Chilled Storage

#### 2.2.1 Sample Treatment

Live sturgeon was washed with sterile water, decapitated, eviscerated, sliced, and rinsed again. After drying of surface moisture, the fillets were vacuum‐packed in sterile bags (about 20 g per bag) and stored at ice temperature (−3°C). Physicochemical properties, sensory indicators, spoilage microbial species, and quantities were analyzed on days 0, 2, 4, 6, 8, 10, 12, and 14.

#### 2.2.2 Microbial Analysis

Modified according to the method of Tan et al. (2022). Under aseptic conditions (SW‐CJ‐10 Ultra‐clean workbench, Suzhou Purification Co. Ltd., Jiangsu, China), 10 g of sturgeon fillets were ground into paste with a mortar, and then poured into 90 mL of sterile saline for 30 min of shaking at 180 r/min. Afterward, 10‐fold gradient dilutions were performed, and the selective media with *aerobic bacteria, Aeromonas*, *Enterobacteria*, and *Pseudomonas counts* were poured into plates by selecting the appropriate dilution factors.

#### 2.2.3 Determination of Total Volatile Basic Nitrogen (TVB‐N)

Chilled sturgeon fillets (20 g) were minced into a paste under sterile conditions, mixed with 100 mL of sterile distilled water in a sealed conical flask, and shaken to ensure uniform dispersion. After soaking for 30 min, the mixture was filtered, and the filtrate was used immediately. Volatile basic nitrogen (VBN) absorbed in boric acid was titrated with a standard solution (Du et al. 2022).

#### 2.2.4 Determination of pH Value

Chilled sturgeon fillets (20 g) were homogenized with 90 mL of distilled water under sterile conditions for 30 min (Gui et al. 2014). The pH was then measured using a pH meter (pHS‐3E pH meter Shanghai Yidian Analytical Instrument Co. Ltd., China.).

#### 2.2.5 Sensory Evaluation

Quality index method (QIM) was used for sensory evaluation. Sturgeon fillet samples stored for different durations were randomly selected and assessed for color, luster, transparency, odor, surface viscosity, and texture. Based on characteristic changes during storage, each sensory attribute was scored on a scale from 0 to 3, where 0 indicated the best quality. A higher score reflected a greater degree of deterioration. The overall sensory quality of the fish was represented by the Quality Index (QI) value, calculated as the sum of the individual parameter scores (Zhang et al. 2022). Table S1 shows the sensory evaluation criteria.

### 2.3 Isolation, Purification and Identification of Dominant Spoilage Bacteria in Chilled Sturgeon Fillets

#### 2.3.1 Preliminary Screening of Spoilage Bacteria

Under aseptic operation, 10 g of deteriorated chilled sturgeon fillets was sampled, minced using sterile scissors, and placed into a sterilized conical flask containing 90 mL of sterile physiological saline. After sealing, the sample was agitated at 180 r/min for 30 min, and a 10‐fold serial dilution was prepared. Three higher dilutions were plated in triplicate on selective media and incubated to isolate spoilage bacteria. Representative colonies were purified by repeated streaking to obtain single colonies. Each was inoculated into 10 mL of nutrient broth and incubated at 30°C or 37°C with shaking for 12–24 h until OD_600_ reached 1.0–1.5. A small aliquot of the culture was collected using a flame‐sterilized inoculating loop and subjected to Gram staining for microscopic examination to confirm purity and detect potential contaminating bacteria. Finally, bacterial suspensions were mixed with 50% sterile glycerol (1:1, v/v), frozen rapidly, and stored at −20°C for later use.

#### 2.3.2 Screening, Identification and Preservation of Specific Spoilage Bacteria

From the 115 spoilage bacterial isolates preliminarily identified from chilled sturgeon fillets, 24 distinct pure strains were selected based on differences in colony morphology, cell shape, and biochemical characteristics. These strains underwent Gram staining and a series of physiological and biochemical tests. The colony morphology—including shape, size, color, edge, surface texture, elevation, and transparency—was carefully documented. Physiological and biochemical assays, such as oxidase, catalase, contactase, glucose and lactose fermentation, Voges‐Proskauer (VP), methyl red (MR), and indole tests, were conducted (Eid et al. 2017).

The species or genera of dominant spoilage bacteria were identified by 16S rDNA sequencing (Kačániová et al. 2019). A total of 24 highly turbid isolates were selected for sequencing. Genomic DNA was extracted using a commercial bacterial DNA extraction kit. The 16S rRNA gene was amplified using universal primers: forward primer 27f (5′‐AGAGTTTGATCCTGGCTCAG‐3′) and reverse primer 1495r (5′‐CTACGGCTACCTTGTTACG‐3′). PCR reactions were performed in a 25 μL mixture containing 12.5 μL 2× T5 Direct PCR Mix, 1 μL of each primer, 1.5 μL template DNA, and 9 μL deionized water. The thermal cycling conditions were: initial denaturation at 95°C for 5 min; 30 cycles of 95°C for 1 min, 55°C for 1 min, and 72°C for 2 min; and a final extension at 72°C for 10 min. Amplification products were confirmed by 1% agarose gel electrophoresis and submitted to Shanghai Shenggong Co. Ltd. for sequencing. The obtained sequences were compared with known sequences in the NCBI database, and isolates showing ≥ 99% similarity were identified. A phylogenetic tree was constructed using the Neighbor‐Joining method in MEGA 5.04.

#### 2.3.3 Spoilage Ability Determination of Dominant Spoilage Bacteria

Seven kinds of spoilage bacteria were inoculated in sterile fish fillets, and spoilage and microbiological indexes were measured during cold storage. The TVB‐N yield factor was calculated by combining the microbial growth parameter: Y_TVB‐N/CFU_ = (TVB‐N content at the spoilage point—TVB‐N content at the initial point)/(bacterial count at the spoilage point—bacterial count at the initial point) (Parlapani et al. 2019).

### 2.4 Sturgeon Fillet Storage by ClO_2_ Bacterial Reduction Treatment

#### 2.4.1 Sample Preparation

*Pseudomonas jessenii* C23, a specific spoilage bacterium isolated from the spoilage endpoint of sturgeon fillets stored in ice and preserved in glycerol tubes, was activated twice in nutrient broth medium. A 2% inoculum was then transferred into fresh nutrient broth and incubated at 30°C for 12 h to obtain a bacterial suspension of 8 log (CFU/mL). The suspension was diluted to 5 log CFU/mL for fillet inoculation. Sterilized white muscle fillets (15–20 g each) were immersed in the bacterial suspension for 15 s, drained, and achieved an initial bacterial load of 4–5 log CFU/mL. Subsequently, the effects of ClO_2_ concentration, immersion time, and fillet thickness on bacterial reduction and residual chlorine content were systematically investigated.

#### 2.4.2 Single Factor Experimental Scheme Design

The single‐factor experimental design is shown in Table S2. Based on the effects of various treatment conditions on the total viable count and the results of the single‐factor tests, three key variables were selected for further optimization following the Box–Behnken design principle: ClO_2_ concentration (20–40 μg/mL), fillet thickness (4–8 mm), and soaking time (10–20 min) (Dowgiałło et al. 2020). A total of 17 experimental runs were generated using Design‐Expert 10.0.3 to construct the response surface model and determine the optimal treatment conditions. The test design was shown in Table S3.

#### 2.4.3 Determination of Inhibition Rate of *Pseudomonas jessenii* C23, a Characteristic Spoilage Bacterium

Sturgeon fillets inoculated with the specific spoilage bacterium *Pseudomonas jessenii* C23 were soaked in ClO_2_ solution at concentrations of 0, 10, 20, 30, 40, and 50 μg/mL for 15 min (solid–liquid ratio 1:20, m/v). After treatment, samples were rinsed with sterile deionized water at 4°C, drained, vacuum‐packed, and stored frozen. Control samples were soaked and rinsed only with sterile deionized water under the same conditions. The bacterial count of *Pseudomonas jessenii* C23 was determined using the plate counting method to evaluate the bactericidal efficacy of ClO_2_ at different concentrations (Yang et al. 2023).

#### 2.4.4 Determination of Water Loss Rate and Residual Chlorine in Sturgeon Fillets

Remove the sample, wipe the surface moisture, record the sample quality before centrifugation, then put filter paper on the bottom of the centrifuge tube, and put the sample on the filter paper at 4°C for centrifugation (4000 r/min, 15 min), record the sample quality after centrifugation (Zhang et al. 2024). Water loss rate (%) = (1—sample mass after centrifugation/sample mass before centrifugation) × 100. The residual chlorine of fish fillets sterilized with ClO_2_ was detected by o‐toluidine colorimetry.

#### 2.4.5 Statistical Analysis

Statistical analyses were performed using SPSS 27 (IL, USA). One‐way ANOVA followed by Duncan's multiple range test was used to compare group means at a significance level of *p* < 0.05. All experiments were conducted in triplicate, and results are presented as mean ± standard deviation. Figures were created using Origin 2024.

## 3 Results and Analyses

### 3.1 Microbial, Physicochemical and Sensory Changes in Sturgeon Fillets During Chilled Storage

#### 3.1.1 Microbial Changes

**FIGURE 1:**
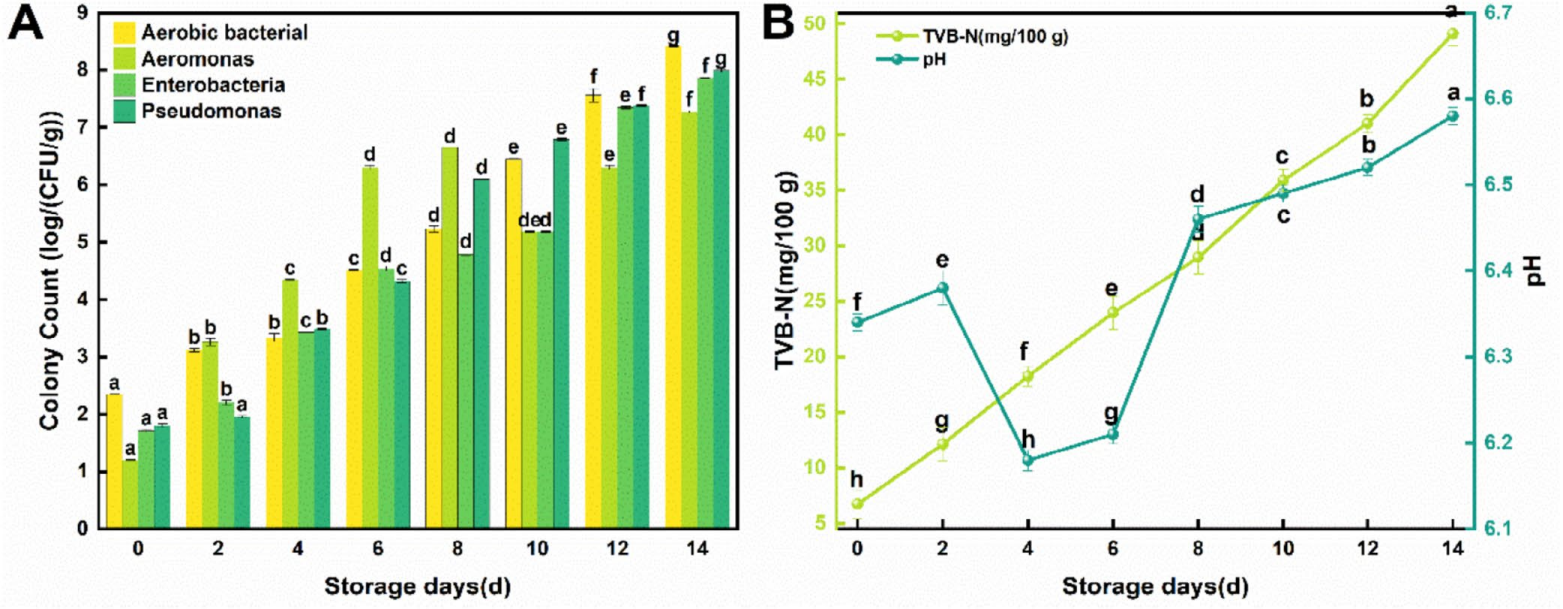
Changes of microbiological (A), TVB‐N, and pH (B) of sturgeon fillets during chilled storage.

Changes in microbial indicators of sturgeon fillets during chilled storage are presented in Figure 1A. The aerobic plate count (APC) showed a continuous upward trend, with all detected microbial counts increasing significantly over the 14‐day storage period (*p* < 0.05). The initial APC was 2.35 log CFU/g, indicating high freshness of the fillets (Sallam 2007). By day 12, the APC reached 6.44 log CFU/g, and by day 14, it exceeded the acceptable limit for raw fish at 7.56 log CFU/g, rendering the fillets unsuitable for consumption (Ojagh et al. 2009). Accordingly, the shelf life of sturgeon samples was 12–13 days. *Aeromonas* and *Pseudomonas* are the main dominant spoilage bacteria of freshwater fish (Sivertsvik et al. 2002). Their counts increased markedly during storage and surpassed the APC by day 4. *Pseudomonas* exhibited a higher growth rate in the later stages, reaching 7.99 log CFU/g at day 14, while *Aeromonas* reached 7.27 log CFU/g. These counts were second only to the APC, confirming their role as dominant spoilage bacteria. The temporal growth patterns suggest that *Pseudomonas* was more active in the early phase, whereas *Aeromonas* dominated later spoilage, consistent with the findings of Bakar et al. (2009). Similarly, Gui et al. reported *Aeromonas* as the dominant spoilage bacterium in vacuum‐packaged chilled sturgeon, supporting our observations (Gui et al. 2014). In addition, Enterobacteria levels increased steadily throughout storage, reaching 7.86 log CFU/g by day 14—only slightly below the counts of *Pseudomonas*. This indicates that Enterobacteriaceae also contributed significantly to spoilage in chilled farmed sturgeon fillets.

#### 3.1.2 TVB‐N

During storage, endogenous enzymes and exogenous microorganisms catalyze the degradation of muscle proteins in fish, producing volatile basic nitrogen compounds such as ammonia, trimethylamine, and dimethylamine. These compounds collectively form TVB‐N, a widely used indicator for assessing fish freshness (Khoa et al. 2024). Figure 1B displays the TVB‐N levels of vacuum‐packaged sturgeon during chilled storage. The initial content of TVB‐N was 6.8 mg/100 g, well below the acceptable limit of 25 mg/100 g. Over time, a marked upward trend in TVB‐N was observed, paralleling microbial growth. This increase results from the synergistic degradation of proteins and non‐protein nitrogenous compounds by endogenous enzymes and spoilage bacteria, generating volatile amines and ammonia. For the first 8 days, the growth rate of TVB‐N was relatively slow. However, from day 8 onward, a rapid increase occurred, reaching 51.4 mg/100 g by day 14, far exceeding the European Commission's spoilage threshold of 25 mg/100 g (Ocaño‐Higuera et al. 2010).

#### 3.1.3 pH

The variation in pH during fish storage is closely associated with microbial metabolism, enzymatic activity, and the accumulation of acidic or alkaline substances, making it a key indicator for assessing fish spoilage. As displayed in Figure 1B, the initial pH of the fish ranged from 6.34 to 6.38, suggesting a state of freshness. After 2–4 days, the pH declined to approximately 6.18, likely due to the production of organic acids by lactic acid bacteria in the early stage of storage. Subsequently, the pH showed an upward trend. This rise in the later stages can be attributed to the onset of autolysis, wherein microbial metabolism leads to the generation of alkaline compounds such as volatile basic nitrogen, trimethylamine, and ammonia (Tan et al. 2022). It is well documented that postmortem glycolysis initially lowers the pH of fish, whereas prolonged storage results in a pH increase due to the degradation of amino compounds through intensified microbial metabolism (Duan et al. 2010; Liu et al. 2013). Throughout the storage process, the pH changed relatively gently, which was associated with the chilled storage environment. Chilled storage could effectively delay microbial growth, resulting in less production of amines.

#### 3.1.4 Sensory Changes

**FIGURE 2:**
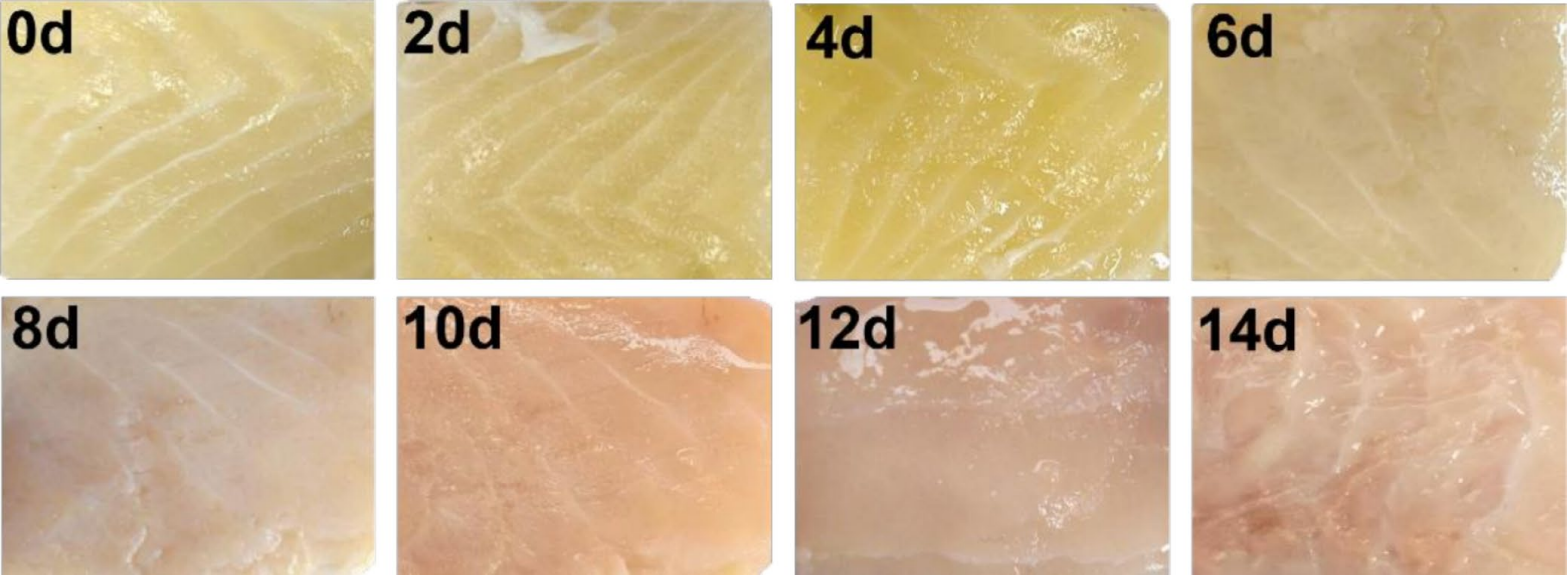
Changes of sturgeon fillets during chilled storage.

QIM sensory indicators provide a direct and intuitive assessment of product quality and reflect consumer acceptance (Anacleto et al. 2019). A higher QI score indicates a gradual decrease in sensory acceptability. As shown in Table 1 and Figure 2, the sturgeon fillets initially exhibited a white, glossy appearance with only a slight fishy odor and flavor. However, all sensory parameters deteriorated significantly over the storage period. By day 4, the fishy odor evidently lightened, the yellow color began to deepen, less mucus appeared, the texture began to soften, and the fillets were still sensorily acceptable. By day 6, various evaluation indicators worsened, and by day 8, the fillets had become seriously sour and rancid, completely yellow, lusterless, and very mucous, which completely reached the sensory rejection point. Meanwhile, during the late stage of storage, the deterioration of fish sensory quality was significantly accelerated. This was primarily linked to the rapid proliferation of spoilage microorganisms, showing consistency with the microbial indicators.

**TABLE 1:** Sensory changes of sturgeon slices during chilled storage.

Time (d)	Color	Luster	Transparency	Odor	Surface viscosity	Texture	QI
0	0.0 ± 0.00^g^	0.0 ± 0.00^f^	0.0 ± 0.00^e^	0.0 ± 0.00^e^	0.0 ± 0.00^g^	0.0 ± 0.00^a^	0.0 ± 0.00^a^
2	0.5 ± 0.04^f^	0.5 ± 0.07^e^	0.5 ± 0.04^d^	1.0 ± 0.05^d^	0.5 ± 0.07^f^	0.5 ± 0.05^d^	3.5 ± 0.02^f^
4	1.0 ± 0.03^e^	1.0 ± 0.02^d^	1.0 ± 0.03^c^	1.5 ± 0.07^c^	1.0 ± 0.04^e^	1.0 ± 0.04^c^	6.5 ± 0.07^e^
6	1.5 ± 0.05^d^	1.5 ± 0.04^c^	1.0 ± 0.05^c^	2.0 ± 0.04^b^	1.5 ± 0.01^d^	1.5 ± 0.06^c^	9.0 ± 0.06^d^
8	2.0 ± 0.07^c^	2.0 ± 0.05^b^	2.0 ± 0.07^b^	2.0 ± 0.03^b^	2.0 ± 0.08^c^	2.0 ± 0.02^b^	12.0 ± 0.04^c^
10	2.0 ± 0.09^c^	2.0 ± 0.01^b^	2.0 ± 0.09^b^	2.0 ± 0.02^b^	2.5 ± 0.06^b^	2.0 ± 0.07^b^	12.5 ± 0.03^c^
12	2.5 ± 0.04^b^	2.0 ± 0.08^b^	3.0 ± 0.04^a^	3.0 ± 0.01^a^	3.0 ± 0.04^a^	3.0 ± 0.01^a^	16.5 ± 0.01^b^
14	3.0 ± 0.01^a^	3.0 ± 0.02^a^	3.0 ± 0.01^a^	3.0 ± 0.03^a^	3.0 ± 0.05^a^	3.0 ± 0.03^a^	18.0 ± 0.01^a^

*Note:* Data are expressed as mean ± standard deviation (SD). Lowercase letters in the same row indicate significant differences (*p* < 0.05).

### 3.2 Isolation, Purification and Preliminary Identification of Dominant Spoilage Bacteria

#### 3.2.1 The Colony Characteristics, Morphological Characteristics and Some Physiological and Biochemical Characteristics of the Isolated Strains Were Studied

During the chilled storage of sturgeon fillets (0–14 days), a total of 115 pure strains were screened from bacterial colonies with typical characteristics on the PCA medium. Following repeated morphological observation and comparison, 24 pure strains differing in colony type, bacterial morphology, and biochemical properties were obtained. The colony and morphological characteristics of the isolated strains are summarized in Table 2. All colonies were round with predominantly smooth and regular margins. Colony surfaces were smooth and glossy, with coloration ranging from white and milky white to golden and pale yellow. Colony size varied among strains, likely reflecting differences in growth rates. Some bacteria exhibited similar growth morphologies and insignificant growth differences. Clearly, the overall differences among these bacteria were not very large, which should be of the same type. According to Table S4, all isolates were rod‐shaped, Gram‐negative bacteria. Most strains could utilize glucose and lactose, were positive for oxidase and catalase activities, and yielded positive results in MR and VP tests. Approximately half of the strains were capable of producing indole.

**TABLE 2:** The characteristics of bacterial colony and morphology of isolated strains.

Strains	Colony shape	Colony color	Colony edges	Colony uplift	Colony surface status	Colony transparency
PL1	Rotundity	golden	Neat	Smooth	Glossy	little transparent
PA17	Rotundity	golden	Neat	Smooth	Glossy	little transparent
2V3	Rotundity	milky white	Neat	Smooth	Glossy	Opaque
2A6	Rotundity	milky white	Neat	Smooth	Glossy	Opaque
2A3	Rotundity	milky white	Neat	Smooth	Glossy	Opaque
2A1	Rotundity	milky white	Neat	Smooth	Glossy	Opaque
2C7	Rotundity	whitish yellow	Neat	Smooth	Glossy	Opaque
2A4	Rotundity	milky white	Neat	Smooth	Glossy	Opaque
2P3	Rotundity	milky white	Neat	Smooth	Glossy	Opaque
C27	Rotundity	whitish yellow	Neat	Smooth	Glossy	Opaque
C23	Rotundity	whitish yellow	Neat	Smooth	Glossy	Opaque
C46	Rotundity	whitish yellow	Neat	Smooth	Glossy	Opaque
C11	Rotundity	whitish yellow	Neat	Smooth	Glossy	Opaque
A21	Rotundity	white	Neat	Smooth	Glossy	Opaque
PA23	Rotundity	whitish yellow	Neat	Smooth	Glossy	Opaque
PL12	Rotundity	milky white	Neat	Smooth	Glossy	Opaque
PA2	Rotundity	whitish yellow	Neat	Smooth	Glossy	Opaque
A18	Rotundity	milky white	Neat	Smooth	Glossy	Opaque
PA3	Rotundity	whitish yellow	Neat	Smooth	Glossy	Opaque
A13	Rotundity	whitish yellow	Neat	Smooth	Glossy	Opaque
PA28	Rotundity	milky white	Neat	Smooth	Glossy	Opaque
PL6	Rotundity	milky white	Neat	Smooth	Glossy	Opaque
A20	Rotundity	milky white	Neat	Smooth	Glossy	Opaque
A19	Rotundity	milky white	Neat	Smooth	Glossy	Opaque

#### 3.2.2 PCR Results for V3 Variable Region of Bacterial 16S rDNAs

The 16S rDNA amplification products of four spoilage bacterial strains isolated from sturgeon fillets stored at ice temperature were analyzed by 1% agarose gel electrophoresis. All strains yielded distinct bands of approximately 1500 bp (Figure S1). As can be seen, the electrophoretic bands were bright and quite clear, indicating that the screened strains were of very high purity, so the amplified 16S rDNAs were also relatively pure and intact, which could satisfy the experimental requirements of the subsequent spoilage ability test and key ClO_2_ bacteriostatic technology exploration.

#### 3.2.3 Sequencing Identification and Phylogenetic Tree Construction

As shown in Table S5, among the 24 strains isolated from samples, *Rahnella aquatilis* was the most dominant, followed by *Pseudomonas* and *Citrobacter*. According to the sequencing results, the major spoilage bacteria in chilled farmed sturgeon fillets were *Rahnella aquatilis* PA23, *Pseudomonas fluorescens* C27, *Pseudomonas jessenii* C23, *Citrobacter freundii* 2A6, *Chryseobacterium indologenes* PL1, *Hafnia alvei* 2C7, and *Serratia fonticola* A19.

Phylogenetic analysis (Figure S2) was performed using sequences with ≥ 99% similarity. By combining the phylogenetic trees with colonial, morphological, physiological, and biochemical characteristics, the dominant spoilage bacteria were primarily classified into *Pseudomonas* and *Enterobacteria*. A19, PL1, and PA17 showed closest affinity to *Chryseobacterium indologenes* of *Enterobacter*. A21, PA23, PL12, PA2, A18, PA3, A13, PA28, PL6, and A20 showed closest affinity to *Rahnella aquatilis* strain of *Enterobacter*. C64, C11, and C23 exhibited closest affinity to *Pseudomonas jessenii* , while C27 displayed closest affinity to *Pseudomonas fluorescens* . 2A4 and 2P3 showed closest affinity to *Klebsiella pneumoniae* ; 2C7 showed closest affinity to *Hafnia alvei* ; 2V3, 2A6, 2A3, and 2A1 showed closest affinity to *Citrobacter freundii* . Thus, the dominant spoilage bacteria could be identified as *Rahnella aquatilis* PA23; *Pseudomonas fluorescens* C27; *Pseudomonas jessenii* C23; *Citrobacter freundii* 2A6; *Chryseobacterium indologenes* PL1; *Hafnia alvei* 2C7; *Serratia fonticola* A19.

### 3.3 Plate Count and TVB‐N Changes in Chilled Farmed Sturgeon Fillets Inoculated With Seven Types of Dominant Spoilage Bacteria

**FIGURE 3:**
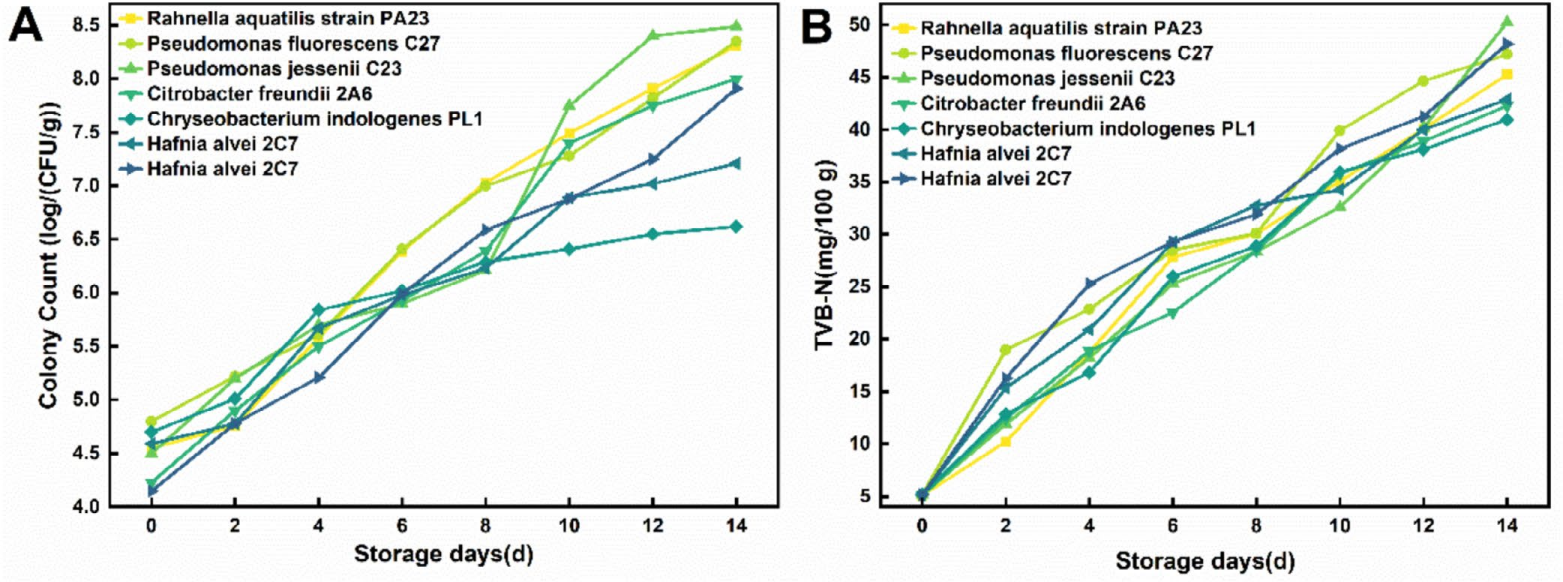
Colony count (A) and TVB‐N (B) changes of predominant corrupt bacteria in chilled breeding sturgeon fillets.

As shown in Figure 3A, the seven dominant spoilage bacteria exhibited continuous growth, and the TVB‐N values also showed a rising trend (Figure 3B). Among them, *Pseudomonas jessenii* C23 exhibited a higher TVB‐N production factor (Y_TVB‐N/CFU_) of 11.29, which was predicated to have the strongest spoilage ability. Thus, *Pseudomonas jessenii* C23 was confirmed as the spoilage bacterium specific to chilled sturgeon fillets. However, other studies have shown that *Aeromonas* is the dominant strain in sturgeon meat. The inconsistency of dominant strains has a great relationship with the growth environment (Gui et al. 2014; Tan et al. 2022).

### 3.4 Single‐Factor Experimental Results and Analysis of ClO_2_ ‐Treated Chilled Sturgeon Fillets

#### 3.4.1 Effect of ClO_2_ Concentration on Inhibition of Characteristic Spoilage Bacteria in Sturgeon Fillets

**FIGURE 4:**
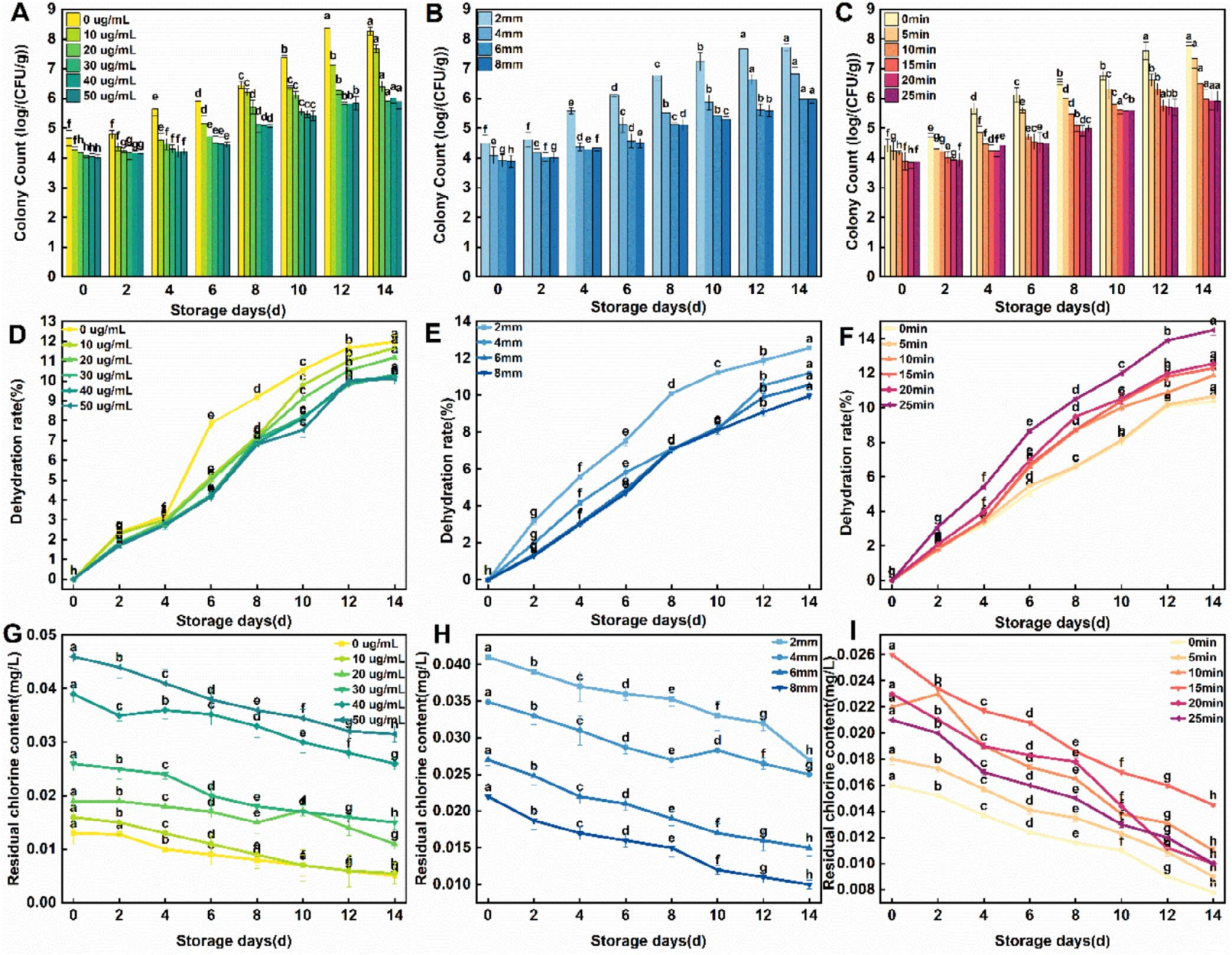
Effects of ClO_2_ concentration, fillet thickness, and soaking time on inhibition of characteristic spoilage bacteria, dehydration rate, and residual chlorine of refrigerated sturgeon fillets.

As shown in Figure 4A, ClO_2_ exhibited a significant inhibitory effect on *Pseudomonas jessenii* C23, a characteristic spoilage bacterium. The inhibitory effect increased with ClO_2_ concentration. In untreated samples, the total viable count reached 6.4 log CFU/g by day 8, whereas samples treated with ClO_2_ maintained a lower count of 6.0 log CFU/g even on day 14. No significant differences in antimicrobial efficacy were observed among ClO_2_ concentrations of 30, 40, and 50 μg/mL. These findings are consistent with those reported by Ji Xiaotong et al. (2018). According to the provisions of the standard for the use of food additives GB 2760–2014, the maximum amount of stable ClO_2_ used in aquatic products and products (fish processing only) is 50 μg/mL. Therefore, the single factor results selected 30 μg/mL use mass concentration in line with the national standard requirements can effectively kill characteristic spoilage bacteria, reduce residual chlorine, lower cost, and improve economic utilization value.

#### 3.4.2 Effect of Sturgeon Fillet Thickness on Inhibition of Characteristic Spoilage Bacteria in Sturgeon Fillets

Sturgeon fillets with thicknesses of 2, 4, 6, and 8 mm were soaked in a 30 μg/mL ClO_2_ solution separately for 15 min, then drained and placed in vacuum bags for chilled storage. The population of *Pseudomonas jessenii* C23, a specific spoilage organism, was quantified via plate counting, as shown in Figure 4B. Fillet thickness had a significant impact on bacterial inhibition, with the 2 mm samples exhibiting the weakest antimicrobial effect. This may be attributable to the small thickness of sturgeon fillets, which led to the increased contact area between fillets and specific spoilage bacterium resulting in its rapid proliferation. The thicker the fillet, the smaller its contact area with specific spoilage bacterium and, accordingly, the lower the bacterial count.

#### 3.4.3 Effect of Soaking Time on Inhibition of Characteristic Spoilage Bacteria in Sturgeon Fillets

Figure 4C illustrates the effects of different soaking durations in ClO_2_ solution on the inhibition of spoilage bacterium in chilled farmed sturgeon fillets. Soaking for 15 min resulted in a *Pseudomonas jessenii* C23 count of 6.01 log CFU/g on day 14, which was significantly lower than in fillets soaked for 0, 5, and 10 min, but not significantly different from those soaked for 20 and 25 min. This outcome may be attributed to a reduction in available chlorine concentration during prolonged soaking. Therefore, 15 min was selected as the optimal soaking time for subsequent experiments.

#### 3.4.4 Effect of ClO_2_ Concentration on the Dehydration Rate of ClO_2_ ‐Treated Chilled Farmed Sturgeon Fillets

Figure 4D displays the effect of ClO_2_ concentration on the dehydration rate of chilled sturgeon fillets. Overall, the dehydration rate increased progressively during storage across all groups. The ClO_2_ concentration produced a certain effect on reducing the fillet dehydration rate, albeit not particularly large.

#### 3.4.5 Effect of Sturgeon Fillet Thickness on the Dehydration Rate of ClO_2_ ‐Treated Chilled Farmed Sturgeon Fillets

Figure 4E illustrates the effect of fillet thicknesses on the dehydration rate of ClO_2_‐treated chilled sturgeon fillets. The dehydration rate of 2‐mm‐thick fillets increased significantly at day 6 and reached a maximum of 12.4% at day 14. Across all thickness groups, the dehydration rates plateaued after day 8, likely due to the maximal loss of free water, followed by a slower rate of bound water loss. Thicker fillets exhibited lower dehydration rates, which may be attributed to their smaller surface area‐to‐volume ratio, as a larger exposed surface area tends to promote higher dehydration.

#### 3.4.6 Effect of Soaking Time on the Dehydration Rate of ClO_2_ ‐Treated Chilled Farmed Sturgeon Fillets

Figure 4F illustrates the effect of soaking duration on residual chlorine in ClO₂‐treated chilled sturgeon fillets, with a constant fillet thickness of 6 mm and the ClO_2_ concentration of 30 μg/mL. The dehydration rate increased with soaking time, reaching 12.2% at 25 min. Across all groups, the dehydration rates leveled off after 12 days, showing consistency with the above analytical conjecture of the fillet thickness effect on dehydration rate.

#### 3.4.7 Effects of ClO_2_ Concentration, Sturgeon Fillet Thickness and Soaking Time on the Residual Chlorine Content in ClO_2_ ‐Treated Chilled Farmed Sturgeon Fillets

Figure 4G–I display the effects of ClO_2_ concentrations, fillet thickness, and soaking time on the residual chlorine content in chilled sturgeon fillets. The residual chlorine content would decrease over storage time due to the decomposition of stable‐state ClO_2_. At the initial stage of storage, the maximum residual chlorine contents were 0.045, 0.041, and 0.018 mg/L, which were completely below the minimum effective dose (1.5 mg) of chlorine on the human body (Malka and Park 2022). Therefore, consumption of the residual chlorine present in the fillets poses no health risk to humans.

### 3.5 Response Surface Design and Result Analysis

#### 3.5.1 Response Surface Design and Results

The total number of colonies optimized by response surface and the experimental design and results of the total number of colonies are shown in Table S6.

Regression analysis results of the colony count model and regression coefficients are detailed in Table S7. Multiple regression analysis was conducted using Design‐Expert 10.0, with ClO_2_ concentration (A), fillet thickness (B), and soaking duration (C) as independent variables, and the total plate count of specific spoilage organisms as the response variable. Table S7 lists the regression model coefficients and significance test results. The following quadratic multinomial regression model was obtained: Y (total plate count of specific spoilage bacterium) = 5.95 − 0.27A − 0.096B − 0.093C + 0.057AB − 0.015AC − 0.13BC + 0.23A^2^ + 0.24B^2^ + 0.16C^2^.

Further regression analysis was performed on the model and its coefficient, with detailed results presented in Table S7. The regression model was highly significant (*p* < 0.01), and the lack of fit was not significant (*p* = 0.14 > 0.05), indicating a good model fit and predictive accuracy. The model exhibited a high coefficient of determination (*R* ^2^ = 0.97; Adj*R* ^2^ = 0.94), suggesting that 93.55% of the variability in the data could be explained by the model, thereby confirming its reliability.

Statistical analysis revealed that the ClO_2_ concentration and fillet thickness had extremely significant effects on the total plate count (*p* < 0.01), while soaking duration showed a significant effect (*p* < 0.05). The main effects ranked in descending order of impact were: ClO_2_ concentration (A) > fillet thickness (B) > soaking duration (C). Among the quadratic interaction terms, BC had a significant effect (*p* < 0.05), whereas AB and AC were not significant (*p* > 0.05). The influence of the interaction terms on total plate count followed the order: BC > AB > AC.

#### 3.5.2 Interactions Among Various Factors

**FIGURE 5:**
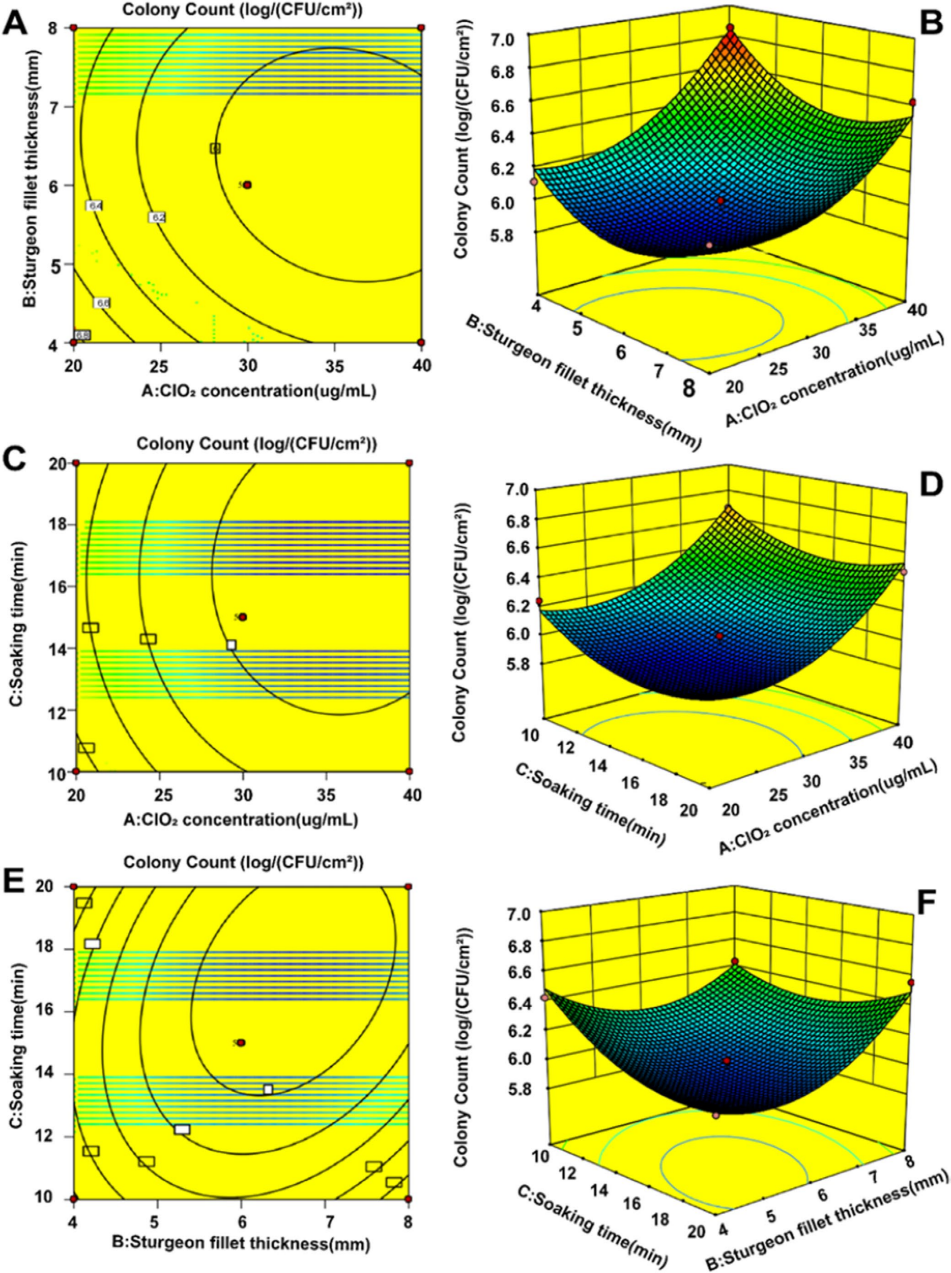
Response surface plot and contour plot.

Based on the regression equation, the response surface curves and contour maps were analyzed to evaluate the effects of ClO_2_ concentration, fillet thickness and soaking duration on the total plate count of specific spoilage bacterium. The response surface curves and contour maps effectively illustrated the interactions among the independent variables. By observing the slope steepness of the response surface curves, the degree of influence of quadratic interaction on the response value was determined. The steeper the curve, the more obvious the interaction. Figure 5A–F illustrate the effects of ClO_2_ concentration (A), sturgeon slice thickness (B) and soaking time (C) on the total plate count of specific spoilage bacterium.

The interactive effects of ClO_2_ concentration and fillet thickness on the total plate count of specific spoilage bacterium are depicted in Figure 5A,B. In the AB interaction surface, the total plate count initially decreased and then increased with increasing fillet thickness, while it gradually decreased and then plateaued with the rising ClO_2_ concentration. The lowest total plate count was observed at ClO_2_ concentrations of 30–40 μg/mL and fillet thickness of 5–7 mm. Notably, the response gradient with ClO_2_ concentration was steeper than that with fillet thickness, suggesting that ClO_2_ concentration exerted a stronger influence. These trends were consistent with the ANOVA results shown in Table S7.

Figure 5C,D illustrates the interactive effects of ClO_2_ concentration and soaking duration on the total plate count of specific spoilage bacterium. In the AC interaction surface, the variation slope of total plate count tended to decrease gradually and then stabilize with the increasing fillet thickness, while it tended to decrease first and then increase slowly with the rising ClO_2_ concentration. The total plate count of specific spoilage bacterium was the smallest when the ClO_2_ concentration was 30–40 μg/mL and the fillet thickness was 14–20 mm. Moreover, the variation steepness of total plate count with ClO_2_ concentration was greater than that with soaking duration, indicating that ClO_2_ concentration had a greater effect on the total plate count than soaking duration. The response surface curve was also consistent with the ANOVA result in Table S7.

Figure 5E,F shows the interactive effect of fillet thickness and soaking duration on the total plate count of specific spoilage bacteria. In the BC interaction surface, the total plate count tended to decrease first and then increase with the prolonging soaking duration. When the soaking time was shorter, the variation slope of total plate count tended to decrease first and then increase with the increasing fillet thickness. When the soaking time was longer, the variation slope of total plate count showed a decreasing trend with the increasing fillet thickness, suggesting a strong interaction between fillet thickness and soaking duration. Moreover, the variation steepness of total plate count with fillet thickness was greater than that with soaking duration, indicating that fillet thickness had a greater effect on the total plate count than soaking duration. The response surface curve was also consistent with the ANOVA result in Table S7.

#### 3.5.3 Verification of Experimental Results

Based on the regression model, the predicted optimal conditions were a ClO_2_ concentration of 35.821 μg/mL, a fillet thickness of 6.48 mm, and a soaking time of 17.08 min. For practical implementation, these parameters were adjusted to a ClO_2_ concentration of 36.00 μg/mL, a fillet thickness of 6.00 mm, and a soaking time of 17 min. Under these optimal conditions, the actual total plate count of specific spoilage bacterium was 5.86 ± 0.65 logCFU/g across three replicates, closely matching the predicted value of 5.84 log CFU/g. This strong agreement between predicted and observed results validates the model's accuracy. Therefore, for industrial processing of chilled farmed sturgeon fillets, optimal inhibition of spoilage bacteria can be achieved by soaking fillets (6–7 mm thick) in a 35–36 μg/mL ClO_2_ solution for 17–18 min.

## 4 Conclusion

By investigating the changes in physicochemical, sensory, and microbiological indicators during chilled storage, we found that farmed sturgeon fillets without ClO_2_ treatment reached the limit values for these parameters at approximately day 10. Seven dominant spoilage bacteria were identified in chilled sturgeon fillets: *Rahnella aquatilis* PA23, *Pseudomonas fluorescens* C27, *Pseudomonas jessenii* C23, *Citrobacter freundii* 2A6, *Chryseobacterium indologenes* PL1, *Hafnia alvei* 2C7, and *Serratia fonticola* A19, among which *Pseudomonas jessenii* C23 was determined to be the specific spoilage organism. Using RSM, the optimal ClO_2_ treatment conditions were determined as a concentration of 36 μg/mL, a fillet thickness of 6 mm, and a soaking time of 17 min. A strong correlation was observed between the predicted and experimental values, demonstrating that these optimized parameters effectively inhibit spoilage bacteria in chilled sturgeon fillets. However, this study has some limitations. Future research will aim to address these issues by conducting comparative studies involving multiple disinfectants and fish species in order to enhance the generalizability and practical relevance of the findings.

## Author Contributions

**Shirui Yu:** data curation (equal), writing – original draft (equal). **Qin Cen:** investigation (equal), resources (equal), validation (equal). **Shan Yu:** methodology (equal), validation (equal). **Wenkang Hu:** conceptualization (equal), software (equal), writing – review and editing (equal). **Xuechun Yin:** formal analysis (equal), visualization (equal). **Min Shan:** supervision (equal). **Xuefeng Zeng:** conceptualization (equal), funding acquisition (equal). **Xiaohua Chen:** project administration (equal).

## Conflicts of Interest

The authors declare no conflicts of interest.

## Supporting information


**Table S1:** Sensory evaluation criteria.
**Table S2:** Single‐factor experimental design table.
**Table S3:** Response surface analysis experimental design table.
**Table S4:** Colony physiological and biochemical characteristics of isolated strains.
**Table S5:** 16SrDNA identification results of 24 strains.
**Table S6:** Response surface optimization of the total number of colonies, the experimental design and results of the total number of colonies.
**Table S7:** Regression analysis results of colony count model and regression coefficien.
**Figure S1:** ElectropHoretic patterns of 16 SrDNA PCR amplification products of 24 strains.
**Figure S2:** Phylogenetic tree of 24 bacterial strains based on 16SrDNA sequence homology.

## Data Availability

All relevant data are included within the manuscript. Any inquiries concerning the findings of this study may be directed to the corresponding author upon request.
