# Correction to “Specific Spoilage Bacterium of Chilled Sturgeon Fillets and the Bacterial Reduction Effect of ClO_2_”

**DOI:** 10.1002/fsn3.71444

**Published:** 2026-01-09

Yu, S., Q. Cen, S. Yu, et al. 2025. “Specific Spoilage Bacterium of Chilled Sturgeon Fillets and the Bacterial Reduction Effect of ClO_2_.” *Food Science & Nutrition* 13, no. 11: e71051. https://doi.org/10.1002/fsn3.71051.

In the originally published version of this article, the funding information was incomplete. The article stated “This research was supported by a subproject of the Agricultural Key Project of the Guizhou Provincial Department of Science and Technology, Qiankehe Support [2019] No. 2328.”

However, an additional funding source that contributed to both the experimental work and publication‐related costs was inadvertently omitted from the Funding section. The Funding statement should be updated to read as follows:

“This research was supported by a subproject of the Agricultural Key Project of the Guizhou Provincial Department of Science and Technology, Qiankehe Support [2019] No. 2328, and the Scientific and Moutai Institute high‐level talents research fund project, mygccrc [2022] 087.”

This correction concerns only the funding acknowledgement and does not affect the data, results, interpretation, or conclusions of the article.

We apologize for this error.

