# Supplementary material for: Specific Spoilage Bacterium of Chilled Sturgeon Fillets and the Bacterial Reduction Effect of ClO2
Source: Food Sci Nutr. 2025 Oct 26;13(11):e71051. doi: 10.1002/fsn3.71051 (PMC12554782; doi:10.1002/fsn3.71051)
Supplement: Supplementary file 1 — Table S1: Sensory evaluation criteria. Table S2: Single‐factor experimental design table. Table S3: Response surface analysis experimental design table. Table S4: Colony physiological and biochemical characteristics of isolated strains. Table S5: 16SrDNA identification results of 24 strains. Table S6: Response surface optimization of the total number of colonies, the experimental design and results of the total number of colonies. Table S7: Regression analysis results of colony count model and regression coefficien. Figure S1: ElectropHoretic patterns of 16 SrDNA PCR amplification products of 24 strains. Figure S2: Phylogenetic tree of 24 bacterial strains based on 16SrDNA sequence homology. [file FSN3-13-e71051-s001.docx]

**Supplementary Material**

**Table S1. Sensory evaluation criteria**

| Grading scale | Quality (sensory score) | | | |
| --- | --- | --- | --- | --- |
|  | 0 | 1 | 2 | 3 |
| Color | Fish white | Begins to turn yellow | Accentuated yellowing | Completely yellow |
| Luster | Lustrous | Ordinary luster | Low luster | No luster |
| Transparency | Transparent | Moderately transparent | Obviously untransparent | Completely untransparent |
| Odor | Light fish flavor | Slightly sour and rancid | Quite sour and rancid | Seriously sour and rancid |
| Surface viscosity | No surface mucus | Less mucous | More mucous | Very mucous |
| Texture | Firm and dense | Rather soft | Soft | Very soft |

**Table S2. Single-factor experimental design table**

| Factor | Level of factor |
| --- | --- |
| ClO_2_ concentration (μg/mL) | 0、10、20、30、40、50 |
| Soaking time (min) | 5、10、15、20、25 |
| Sturgeon slice thickness (mm) | 2、4、6、8 |

**Table S3. Response surface analysis experimental design table**

|  | |  | Factor |  |
| --- | --- | --- | --- | --- |
| Level | | A- ClO_2_ concentration (μg/mL) | B- Sturgeon slice thickness (min) | C- Soaking time (min) |
| -1 | 20 | | 4 | 10 |
| 0 | 30 | | 6 | 15 |
| 1 | 40 | | 8 | 20 |

**Table S4. Colony physiological and biochemical characteristics of isolated strains**

| Strains | Cell morphology | Gram staining | Enzyme contact test | Catalase test | Glucose | Lactose | Voges-Proskauer | Methyl red | Indole |
| --- | --- | --- | --- | --- | --- | --- | --- | --- | --- |
| PL1 | God-shaped | - | + | + | + | + | + | + | - |
| PA17 | God-shaped | - | + | + | + | + | + | + | - |
| 2V3 | God-shaped | - | - | + | + | + | + | + | - |
| 2A6 | God-shaped | - | - | + | + | + | + | + | - |
| 2A3 | God-shaped | - | - | + | + | + | + | + | - |
| 2A1 | God-shaped | - | - | + | + | + | + | + | - |
| 2C7 | God-shaped | - | - | + | + | + | + | + | - |
| 2A4 | Short rod-shaped | - | - | + | + | + | + | + | + |
| 2P3 | Short rod-shaped | - | - | + | + | + | + | + | + |
| C27 | Short rod-shaped | - | + | + | + | + | - | - | + |
| C23 | Long rod-shaped | - | + | + | + | - | - | - | - |
| C46 | Short rod-shaped | - | + | + | + | + | - | - | + |
| C11 | Long rod-shaped | - | + | + | + | - | - | - | - |
| A21 | God-shaped | - | + | + | + | + | + | + | + |
| PA23 | God-shaped | - | + | + | + | + | + | + | + |
| PL12 | God-shaped | - | + | + | + | + | + | + | + |
| PA2 | God-shaped | - | + | + | + | + | + | + | + |
| A18 | God-shaped | - | + | + | + | + | + | + | + |
| PA3 | God-shaped | - | + | + | + | + | + | + | + |
| A13 | God-shaped | - | + | + | + | + | + | + | + |
| PA28 | God-shaped | - | + | + | + | + | + | + | + |
| PL6 | God-shaped | - | + | + | + | + | + | + | + |
| A20 | God-shaped | - | + | + | + | + | + | + | + |
| A19 | God-shaped | - | + | + | + | + | - | + | + |

**Table S5. 16SrDNA identification results of 24 strains**

| Strains | The most similar strains in NCBI | Login Number | Homology |
| --- | --- | --- | --- |
| PL1 | *Chryseobacterium indologenes strain* CI13 | MT793136.1 | 99.57% |
| PA17 | *Chryseobacterium indologenes strain*FDAARGOS_510 | CP033828.1 | 99.06% |
| 2V3 | *Citrobacter braakii strain* FDAARGOS | CP077300.1 | 99.58% |
| 2A6 | *Citrobacter freundii strain* 261-bpink | MN208068.1 | 99.86% |
| 2A3 | *Citrobacter freundii strain*Ateta | KF245926.1 | 99.58% |
| 2A1 | *Citrobacter freundii strain*UMH19 | CP024673.1 | 99.79% |
| 2C7 | *Hafnia alvei strain*I2 | KT767797.1 | 99.58% |
| 2A4 | *Klebsiella pneumonia strain*1 | MZ389269.1 | 99.79% |
| 2P3 | *Klebsiella pneumonia strain*1 | MZ389269.1 | 99.79% |
| C27 | *Pseudomonas fluorescens strain*Y37B | MW295495.1 | 99.35% |
| C23 | *Pseudomonas jessenii strain*CeD-5 | MN220616.1 | 100.00% |
| C46 | *Pseudomonas jesseniis train*CeD-5 | MN220616.1 | 99.71% |
| C11 | *Pseudomonas jessenii strain*JM7 | MN758767.1 | 99.56% |
| A21 | *Rahnella aquatilis strain*DGE4 | MK764975.1 | 99.79% |
| PA23 | *Rahnella aquatilis strainDGE5* | MK764976.1 | 99.93% |
| PL12 | *Rahnella aquatilis strain*DGE5 | MK764976.1 | 99.86% |
| PA2 | *Rahnella aquatilis strain*IAE75 | MK415023.1 | 99.72% |
| A18 | *Rahnella aquatilis strain*LZH-G10 | OL687518.1 | 99.72% |
| PA3 | *Rahnella aquatilis strain*YL-245 | OK136241.1 | 99.72% |
| A13 | *Rahnella aquatilis strain*YL-245 | OK136241.1 | 99.72% |
| PA28 | *Rahnella aquatilis strain*YL-245 | OK136241.1 | 99.79% |
| PL6 | *Rahnella aquatilis strain*YL-255 | OK136244.1 | 99.58% |
| A20 | *Rahnella inusitata strain*VT25B | MK100816.1 | 99.65% |
| A19 | *Serratia fonticola strain*BXC25 | MN227497.1 | 98.69% |

**Table S6. Response surface optimization of the total number of colonies, the experimental design and results of the total number of colonies**

| Test number | ClO_2_ concentration（μg/mL） | Sturgeon slice thickness（mm） | ClO_2_ concentration（min） | Colony Count  （log CFU/g） |
| --- | --- | --- | --- | --- |
| 1 | 20 | 4 | 15 | 6.85 |
| 2 | 40 | 4 | 15 | 6.11 |
| 3 | 20 | 8 | 15 | 6.62 |
| 4 | 40 | 8 | 15 | 6.11 |
| 5 | 20 | 6 | 10 | 6.67 |
| 6 | 40 | 6 | 10 | 6.24 |
| 7 | 20 | 6 | 20 | 6.47 |
| 8 | 40 | 6 | 20 | 5.98 |
| 9 | 30 | 4 | 10 | 6.43 |
| 10 | 30 | 8 | 10 | 6.42 |
| 11 | 30 | 4 | 20 | 6.55 |
| 12 | 30 | 8 | 20 | 6.02 |
| 13 | 30 | 6 | 15 | 6.01 |
| 14 | 30 | 6 | 15 | 5.89 |
| 15 | 30 | 6 | 15 | 5.95 |
| 16 | 30 | 6 | 15 | 5.91 |
| 17 | 30 | 6 | 15 | 6.01 |

**Table S7. Regression analysis results of colony count model and regression coefficien**

| Source | Sum of squared deviations | Freedom | Mean square | F value | P value | Significance |
| --- | --- | --- | --- | --- | --- | --- |
| Model | 1.45 | 9 | 0.16 | 26.79 | 0. 01 | ** |
| A-ClO_2_ concentration | 0.59 | 1 | 0.59 | 98.11 | <0.01 | ** |
| B-Sturgeon slice thickness | 0.074 | 1 | 0.074 | 12.35 | 0.0098 | ** |
| C-Soaking time | 0.068 | 1 | 0.068 | 11.41 | 0.0118 | * |
| AB | 0.013 | 1 | 0.013 | 2.2 | 0.1812 |  |
| AC | 9.00E-04 | 1 | 9.00E-04 | 0.15 | 0.71 |  |
| BC | 0.068 | 1 | 0.068 | 11.27 | 0.0121 | * |
| A2 | 0.22 | 1 | 0.22 | 36.09 | 0.0005 | ** |
| B2 | 0.25 | 1 | 0.25 | 41.02 | 0.0004 | ** |
| C2 | 0.11 | 1 | 0.11 | 17.8 | 0.0039 | ** |
| Residual | 0.042 | 7 | 6.00E-03 |  |  |  |
| Omitted variable | 0.03 | 3 | 9.89E-03 | 3.21 | 0.1446 | ns |
| Pure error | 0.012 | 4 | 3.08E-03 |  |  |  |
| Sum | 1.49 | 16 | |  |  |  |
|  | R^2^=0.97 AdjR^2^=0.94 | | |  |  |  |

*p* < 0.01 is extremely significant and is represented by **; *p* < 0.05 is significant and is represented by *; *p* > 0.05 is not significant and is represented by ns.

**
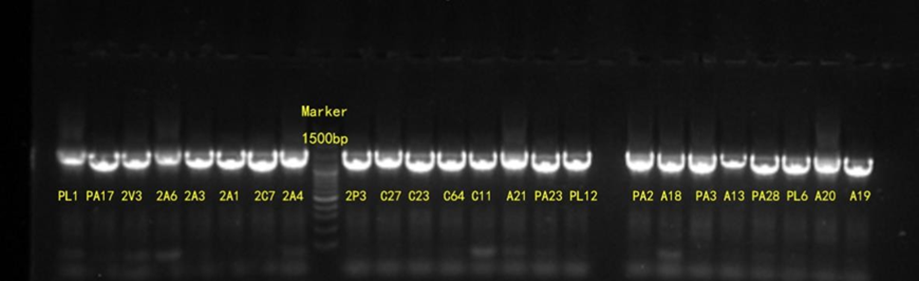
**

Fig. S1. ElectropHoretic patterns of 16 SrDNA PCR amplification products of 24 strains.

**
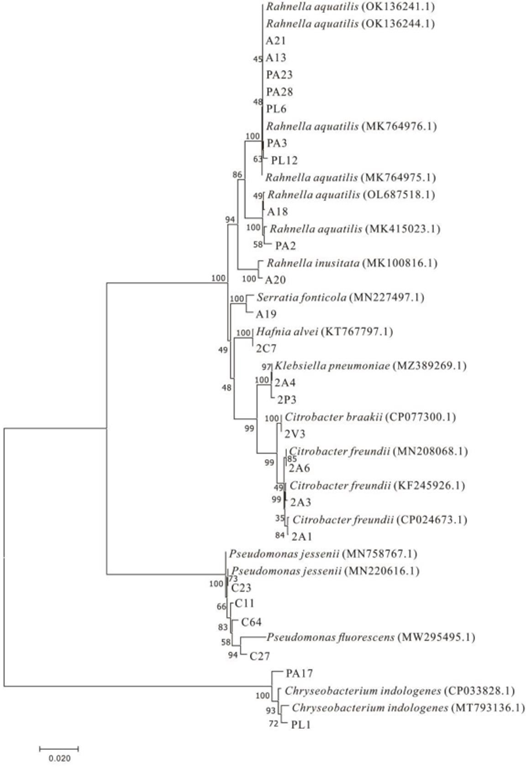
**

**Fig. S2.** Phylogenetic tree of 24 bacterial strains based on 16SrDNA sequence homology
